# Cleavage of Early Mouse Embryo with Damaged DNA

**DOI:** 10.3390/ijms23073516

**Published:** 2022-03-23

**Authors:** Vladimír Baran, Tomáš Ďuríček, Jozef Pisko, Dávid Drutovič, Petr Šolc

**Affiliations:** 1 Institute of Animal Physiology, Centre of Biosciences, Slovak Academy of Sciences, Šoltésovej 4, 040 00 Košice, Slovakia; pisko@saske.sk; 2 Institute of Animal Physiology and Genetics of the Czech Academy of Sciences, Rumburská 89, 277 21 Liběchov, Czech Republic; tom.duricek@gmail.com (T.Ď.); drutovic@iapg.cas.cz (D.D.); 3 Department of Cell Biology, Faculty of Science, Charles University, Viničná 7, 128 00 Prague, Czech Republic

**Keywords:** mouse embryogenesis, DNA damage, neocarzinostatin, γH2A.X, micronucleus

## Abstract

The preimplantation period of embryogenesis is crucial during mammalian ontogenesis. During this period, the mitotic cycles are initiated, the embryonic genome is activated, and the primary differentiation of embryonic cells occurs. All cellular abnormalities occurring in this period are the primary cause of fetal developmental disorders. DNA damage is a serious cause of developmental failure. In the context of DNA damage response on the cellular level, we analyzed the course of embryogenesis and phenotypic changes during the cleavage of a preimplantation embryo. Our results document that DNA damage induced before the resumption of DNA synthesis in a zygote can significantly affect the preimplantation development of the embryo. This developmental ability is related to the level of the DNA damage. We showed that one-cell embryos can correct the first cleavage cycle despite low DNA damage and incomplete replication. It seems that the phenomenon creates a predisposition to a segregation disorder of condensed chromatin that results in the formation of micronuclei in the developmental stages following the first cleavage. We conclude that zygote tolerates a certain degree of DNA damage and considers its priority to complete the first cleavage stage and continue embryogenesis as far as possible.

## 1. Introduction

The primary role of an early embryo is the resumption of mitotic activity and the initiation of the expression of the embryonic genome. This will ensure the correct transfer of genetic information to the new generation of the species. The control of the maintenance of genomic integrity is important for all cell types but for early embryonic cells it is crucial. Failure of the control machinery leads to the elimination (or uncontrolled proliferation) of these somatic cells within tissues. Failure of the control mechanisms in the preimplantation embryo has very serious overall consequences for the overall development of further embryogenesis and to long-term effects on offspring [1]. From this aspect, early embryos overcome DNA damage using a different “strategy” compared with somatic cells [2]. Although all the consequences of DNA lesions generated during the reprogramming are not well-known, it is known that one-cell embryos must maintain genomic stability during the long-running cell cycle period. During the early stages of a preimplantation embryo, dynamic epigenetic modification of the genome occurs [3]. In this context, DNA demethylation can be observed. These phases of DNA demethylation are concomitant with the appearance of DNA strand breaks and DNA repair markers such as γH2A.X and PARP-1, respectively [4]. In principle, the repair can result in three possible outcomes: (i) the correction of the damage as the best option, (ii) the activation of the apoptotic pathway, leading to cell death, and (iii) tolerance of the lesion, which can lead to mutation or eventual carcinogenesis [5]. In further studies, it was suggested that the dominant response to DNA damage in such embryogenesis was DNA repair rather than cell division (tolerance of the lesions) or apoptosis [6]. Immediately after fertilization, sperm chromatin undergoes a massive reorganization during which the protamines are exchanged for maternal nucleosomes. The DNA lesions are generated during paternal DNA demethylation and repaired during the first cell cycle after fertilization to prevent chromosome fragmentation, embryo loss, and infertility [7]. Additionally, these DNA lesions followed by correct repair create the conditions for genome diversity in response to endogenous/exogenous causes. On the other hand, the initial embryonic cell cycles are characterized by a higher degree of genome instability, which can cause congenital disorders and most of them are due to chromosomal abnormalities [8]. As estimated, about half of blastocysts appear to contain genomic alterations that cause a high incidence of pregnancy losses [9]. In this context, the signaling pathways induced by DNA damage during the early stages of embryo development are crucial for the activation of downstream effectors that control the integrity of genomic DNA. In addition, the involvement of other DNA repair pathways, such as homologous recombination in addition to “BER repair” during zygotic reprogramming, is still hypothetical. Generally, cell cycle control machinery is represented by three major checkpoints that are utilized to ensure the progression of the cell cycle. These include checkpoints G1/S, G2/M, and SAC. The G1/S checkpoint is extremely sensitive to the DNA damage response pathway. The activation of the control point can inhibit the activation of Cdk2 or Cdk1 and prevent S-phase entry as well as DNA replication. The G2/M checkpoint represents the final control point of the cell cycle before entry into mitosis. The activation of this control point is influenced by upstream inputs and acts to stall the cell in the G2 stage if some chromosomal aberrations are detected. The spindle assembly checkpoint (SAC) represents the major control point in the phase onset of cytokinesis. It is activated during the metaphase—anaphase transition and prevents the premature separation of sister chromatids during the prometaphase by delaying the onset of the anaphase [10]. The cell cycle checkpoints are primarily and differentially regulated by ATM and ATR in response to genotoxic stress, and they act as initial points for the containment of genomic damage. Under conditions of extensive or persistent DNA damage, the demise of the embryo is the ultimate method of protecting genomic integrity [11]. On the other hand, the precise replication of the genome during the S-phase is of fundamental importance, especially in one-cell embryos. Double-stranded breakage is probably the most serious type of DNA damage as it induces chromosomal instability and failed rearrangements [5]. DNA double-stranded and single-stranded breaks are marked by phosphorylated histone H2A.X, designated as γH2A.X^S139^ [12]. As was earlier presented, shortly after gamete fusion the γH2A.X signaling pathway is activated during sperm chromatin remodeling in a dose-dependent manner, reflecting the number of DNA breaks immediately after various in vivo male irradiations [13]. Remarkably, γH2A.X foci are detected at the time of paternal DNA demethylation [4], suggesting that DNA breaks are indeed generated during zygotic reprogramming. With this understanding, a role of γH2A.X signaling in the zygote for both male and female chromatin can be expected shortly after fertilization, as aconsequence of male chromatin remodeling. It was shown earlier that DNA damage in oocyte, zygote or early embryo induced by γ-irradiation or laser microbeams [14,15,16], and chemical drugs such as etoposide, bleomycin or neocarzinostatin [13,16,17,18], results in cleavage delay. Thus, damaged cells can complete the cell cycle but have a tendency to increase the micronuclei formation. In the case of cleaving embryos, development is compromised in the next cleavage and very often is arrested before the blastocyst stage. The application of other DNA damage inductors updated knowledge in this area. The UV irradiation of two-cell embryos caused G2/M arrest and cisplatin (*cis-diammineplatinum(II)dichloride*) inactivation of the G1/S checkpoint [11]. The delay of DNA replication induced by sperm DNA damaged results in retardation during progression into the two-cell stage followed by the arrest of a large portion of the embryos at the G2/M border [19].

In our previous study we demonstrated that oocytes containing a mean degree of DNA damage are able to finish maturation in spite of an increased number of lagging chromosomes emerging in anaphase-I, resulting in chromosomal fragment cell phenotypes at the end of oocyte maturation (metaphase II) [18]. These results confirm earlier observations that DNA damage in fully grown oocytes does not activate the G2/M checkpoint and allows progression in meiosis I. On the other hand, oocytes that have a high degree of DNA damage fail to complete meiosis [20]. Finally, there is limited knowledge about the activity and “effectiveness” of cell cycle checkpoints in oocytes as well as early embryos. In older images, the checkpoint mechanisms are not fully functional in very early embryos because the time to repair DNA damage is insufficient during rapid cleaving. Consequently, the embryo will cleave incorrectly and finally die before implantation [21]. Although a few studies have dealt with cell phenotypes of early embryos derived from DNA-damaged germinal cells [19,22], there is a lack knowledge on the developmental consequences of preimplantation embryos in which DNA was damaged before the resumption of the first mitotic activity. Sub-cellular abnormalities often occurring after in vitro fertilization have been linked to DNA damage, which is considered to be the primary cause of a decline in embryonic developmental success [23]. In this study, we focused on the development of mouse preimplantation embryo in which DNA damage was induced before the S-phase of the one-cell stage.

## 2. Results

### 2.1. Incorporation of EdU into DNA in Individual Cell Cycle Phases of One-Cell Embryos

**Figure 1:**
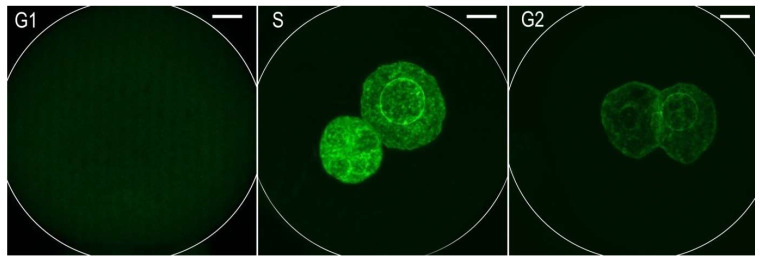
Immunofluorescence labeling the of5-ethynyl-2’-deoxyuridine (EdU) incorporated into DNA separately during the G1-phase, S-phase, or G2-phase in one-cell mouse embryos. The Z-projection of the confocal sections across the whole pronuclei is shown. Scale bar = 10 µm.

Although individual phases during the first cell cycle of mouse embryos can be relatively well-distinguished based on time intervals after hCG administration, we used an EdU assay that detects the incorporation of ethynyl-deoxyuridine into DNA during active DNA synthesis. Thus, the dynamic metabolic labeling of DNA documented the level of DNA replication activity during the exposure of one-cell embryos to 5-ethynyl-2’-deoxyuridine (EdU). The culture medium was supplemented with EdU at 19 h post hCG administration (G1-phase)for 30 min. When the culture medium was supplemented with EdU at 23 h post hCG administration (S-phase) for 30 min, both the male and female pronuclei of one-cell embryos were strongly immunopositive on the incorporation of uridine into DNA. When the culture medium was supplemented with EdU at 30 h post hCG administration (G2-phase) for 30 min, the immunosignal over the male and female pronuclei of one-cell embryos was very weak (Figure 1). Based on this experiment, we determined a more accurate timing for of radiomimetic drug neocarzinostatin (NCS) in the G1-phase within our mouse model in relation to hCG administration. The immunofluorescence signal showed that DNA replication activity was not completely negative in the G2-phase.

### 2.2. Neocarzinostatin Increasesthe Incidence of yH2AX^Ser139^-Positive Foci during the First Embryonic Cell Cycle

**Figure 2:**
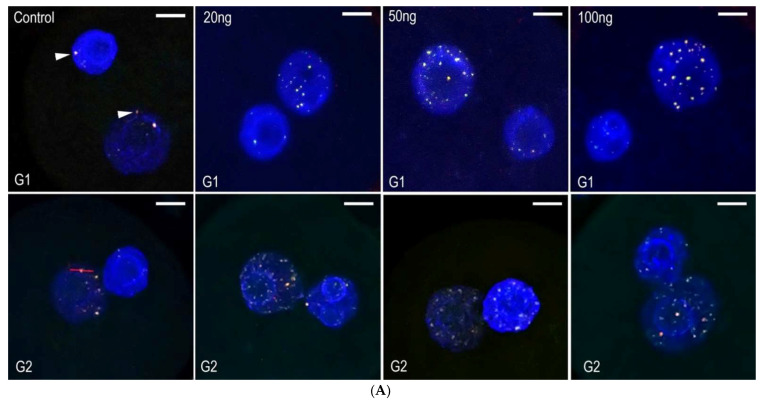

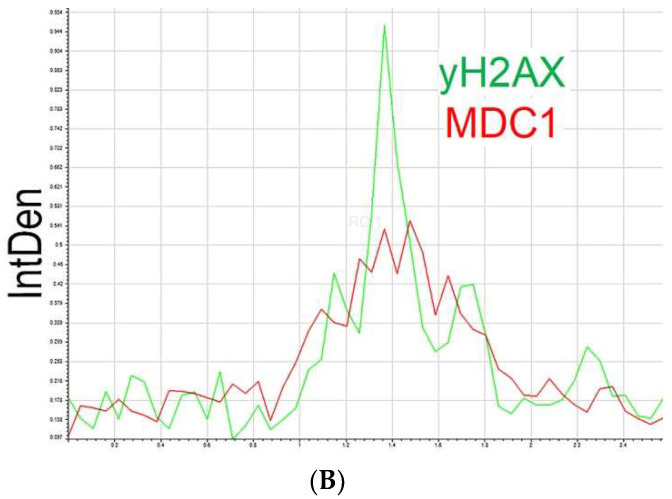
(**A**) Double immunostaining of γH2AX^Ser139^/MDC1 (arrowheads) in pronuclei during the G1- and G2-phases of one-cell stage embryos. The DAPI staining (blue) shows pronuclei. The Z-projections of confocal section across whole nuclei are shown. Scale bar = 10 µm. (**B**) Graph represents the fluorescence image profile and co-localization analyses for γH2AXSer139 /MDC1 in the immunopositive foci. The *x*-axis represents the length of analyzed line on the picture “Control G2” (red bar).

**Figure 3:**
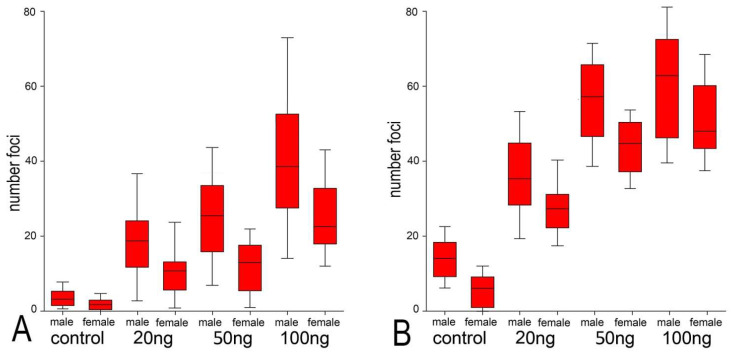
Quantification of γH2AX^Ser139^/ MDC1 immunopositive foci in the male or female pronuclei of control and NCS-treated one-cell embryos analyzed in the G1-phase (**A**) and G2-phase (**B**). The values show the average number of foci in the one-cell embryos of each experimental group.

**Figure 4:**
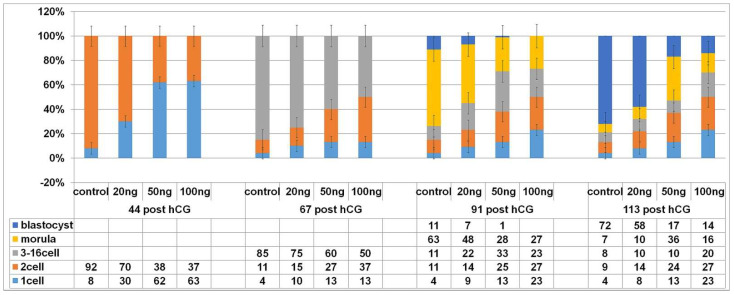
Cleavage ability of control and NCS-treated embryos in in vitro conditions. The data show a percentage of cleavage stages in the individual experimental groups at the periods after hCG administration. Total numbers of embryos examined in each group: control = 112; 20 ng NCS = 135; 50 ng NCS= 140; 100 ng NCS= 145. The experiment was repeated three times.

The radiomimetic neocarzinostatin (NCS), used in this study, has long been identified as a highly potent DNA damaging agent. The non-proteinaceous component (chromophore) of this drug is highly active in the minor groove of DNA, where sequence-specific breaks are induced [24]. One-cell mouse embryos were exposed to three different concentrations of NCS (20, 50, and 100 ng/mL), and then, the number of yH2AX^Ser139^-positive foci was analyzed in the male and female pronuclei of these embryos. We detected the colocalization of phosphorylated histone γH2A.X^Ser139^ and MDC1 protein, which interact with near sites of DNA double-stranded breaks (Figure 2A, arrowheads). These immuno-positive foci were distributed in the whole volume of both the male and female pronuclei. Double immunostaining and spectral analysis of γH2A.X^Ser139^ and MDC1 immunosignals revealed their mutual colocalization and confirmed the location of γH2A.X^Ser139^ on the DNA break sites (Figure 3B). Immediately after the NCS treatment (in the G1-phase), the number of γH2A.X^Ser139^/MDC1-positive foci was increased in comparison with control embryos. The increase in the number of foci was related to the increased NCS concentration. During the G2-phase, an increased number of foci were detected in control embryos compared with the control group in the G1-phase. In the NSC-treated embryos, an increased number of yH2AX^Ser139^/MDC1-positive foci in the G2-phase were detected compared with the same groups in the G1-phase, being NCS concentration-dependent. In the G2-phase, the increase in foci was significantly higher in NSC-treated embryos compared with the G2-phase control embryos (Figure 4). In summary, these data demonstrate that NCS induces DNA double-stranded breaks even after short treatment in early embryos cultured in vitro. The double-stranded breaks were well-detectable and well-quantifiable in early mouse embryos.

### 2.3. DNA Damage Reduce the Cleavage Ability of Early Embryos

In a previous experiment we demonstrated that NCS induces DNA double-stranded breaks (DNA damage). This drug was applied in the G1-phase of the first cleavage stage to assess whether it affects the subsequent development of the embryo. The progression of zygotic development following induced DNA damage before the first S-phase after fertilization is shown in Figure 4.

At the time interval of 44 h post hCG administration, 92% of the control embryos completed their first cleavage. Such embryos displayed the two-cell stage. At the same time point, approximately two-thirds (70%) of the embryos treated with 20 ng/mL NCS completed their first mitosis, and the other (30%) were still at the one-cell stage. In higher concentration of NCS (50 or 100 ng/mL), 38/37% were cleaved into the two-cell stage and 62/63% were still at the one-cell stage.

At the time interval of 67 h post hCG, 85% of the control embryos displayed the three-cell up to the sixteen-cell stage and 11% only displayed the two-cell stage. At the same time (67 h post hCG), the embryos treated with a lower concentration of NCS (20 ng/mL) were lightly delayed in cleavage ability. Of the embryos, 75% reached the three-cell up to the sixteen-cell stage, and a quarter of these treated embryos were still in the two-cell stage (15%) or arrested in the one-cell stage (10%). In the same period, only 60% or 50% of the embryos treated with higher NCS concentrations (50 or 100 ng/mL) were cleaved into the three-cell up sixteen-cell stage. In both groups, approximately one-third of the embryos were still in the two-cell stage, and 13% were arrested in the first cleaving stage.

At the time interval of 91 h post hCG, 11% of the control embryos reached the blastocyst stage, 63% displayed the compact multicellular morula stage, 11% did not exceed the sixteen-cell stage, and a further 11% the two-cell stage. In the same period, only 7% of the embryos treated with 20 ng/mL NCS developed into blastocysts and approximately half of the embryos of the group (48%) were still in the compact multicellular morula or lower developmental stages 22% did not exceed the sixteen-cell stage and 14% remained in the two-cell stage. By the same time interval, no embryo treated with a higher dose of NCS (50 or 100 ng/mL) reached more than the compact morula stage, with the exception of 1% of the group treated with 50 ng/mL NCS. Approximately one-third of the embryos (in higher NCS concentrations) reached the compact morula stage (28 and 27%); additionally, approximately one-third was still in the two-cell stage (25 and 27%). In the case of the evaluation of the embryos containing three to sixteen-cell cells, more (33%) were calculated in the 50 ng NCS group, with 23% in the 100 ng NCS group.

At the time interval of 113 h post hCG, nearly three-thirds (72%) of the control embryos reached the expanded blastocyst stage and only 7% reached the compact morula stage. In this group. A total of17% of the embryos were arrested in lower stages, 8% reached three-sixteen-cells, and 9% reached only two-cells stage. After a lower dose of NCS (20 ng/mL) in the one-cell stage, more than half of the embryos (58%) were able to develop into blastocysts, only 10% were still in the compact morula stage, and a further 10% were in stages displaying three-sixteen cells. Of the thus treated embryos, 14% were arrested in the two-cell stage. The higher doses of NCS (50 or 100 ng/mL) limited embryo development because only 17% or 14% of them reached the blastocyst stage. In the case of a concentration of 50 ng/mL, more than one-third (36%) of the embryos were in the compact morula stage, in comparison with 16% at a higher NCS concentration (100 ng/mL). The evaluation of lower embryo stages showed higher numbers of arrested two-cell embryos (24% and 27%) compared with three-sixteen-cell embryos (10% and 20%).

Individual numerical data and their mutual relations at the time interval of 138 h post hCG are comparable with the previous time interval (113 h post hCG). Only very small insignificant changes in the number of blastomeres were noted (not shown in the table presented in Figure 4).

The fertilized but non-cleaving one-cell embryos were arrested predominantly in the pronuclear phase in the control as well as NCS-treated groups. Taken together, these data suggest that DNA damage induced in one-cell embryos before the resumption of DNA synthesis slows the development and gradually reduces the cleavage ability of the mouse embryo in relation to the level of DNA damage.

### 2.4. Higher DNA Damage Level Cause Decreased Volume of DNA after the End of the S-Phase

**Figure 5:**
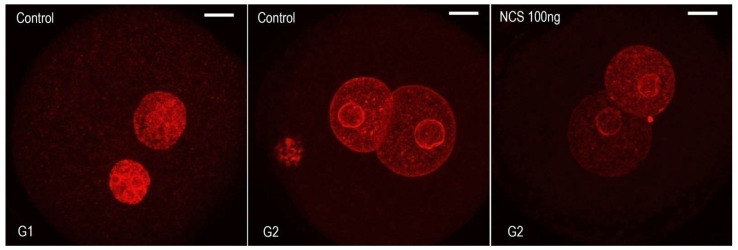
Propidium iodide staining of pronuclei in control (in the G1- vs.the G2-phase) and NCS-treated (in the G2-phase) one-cell embryos. The Z-projection of the confocal sections across the whole pronuclei is shown. Scale bar =10 µm.

**Figure 6:**
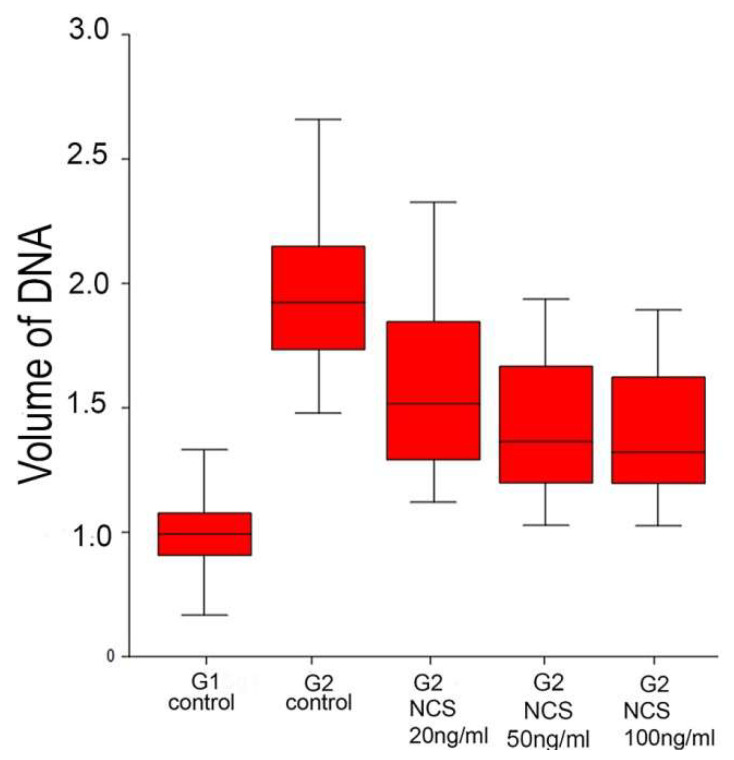
Quantification of the total volume of DNA in control and NCS-treated one-cell embryos. The graph shows the relativized values of the measured fluorescent signal densities.

Our experiments using the incorporation of ethynyl-deoxyuridine into DNA, indicated that DNA synthesis can continue beyond the usual period of the S-phase (to the expected time of the G2-phase) when the embryos were previously treated with NCS (Figure 5). DNA staining using propidium iodide methods followed with morphometric analysis of the serial confocal sections provided precise data about the volume of DNA in the pronuclei. Measurement and relativized data on the volume of DNA in the G2-phase showed the differences between the control embryos and neocarzinostatin-treated embryos. The embryos treated with three different concentrations of neocarzinostatin (20, 50, and 100 ng/mL) during the G1-phase displayed lower volumes of DNA in the pronuclei. Thus, the reduced volumes of DNA weredependent on the concentration of the drug (Figure 6).

### 2.5. DNA Damage CanInduce Chromosomal Missegregation

**Figure 7:**
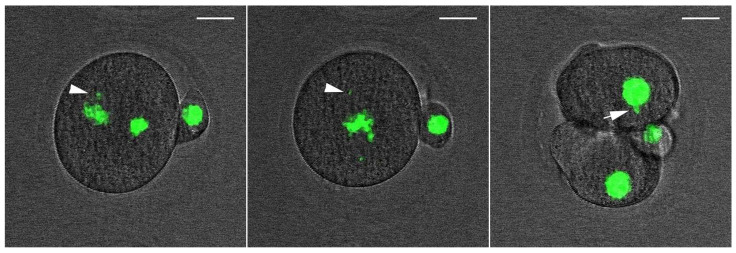
Still images form a time-lapse recording of one-cell mouse embryo expressing H2B-GFP during the first mitosis. The misalignment of chromatin is visible before the cleavage process (arrowheads) as is a micronuclear body in one blastomere of a two-cell embryo (arrow). Additionally, see Movie S1 in the Supplementary Data. Scale bar = 2 µm.

**Figure 8:**
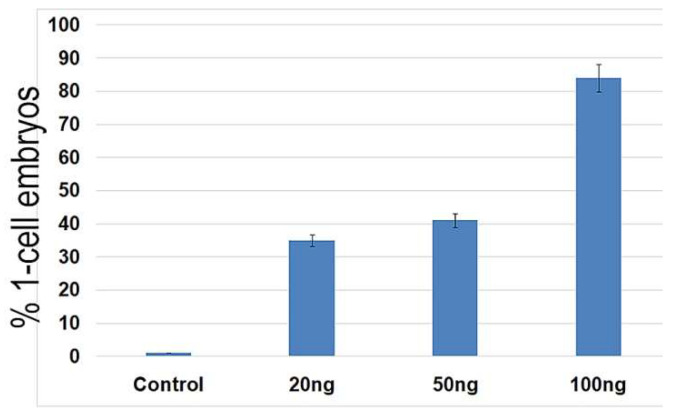
Percentage of the control and NCS-treated one-cell embryos they contain chromosome segregation errors.

For monitoring the progress of nuclear envelope breakdown (NEBD), the chromosomes alignment during metaphase, and the anaphase in the one-cell embryo stage and during the formation of nuclei in the early two-cell stage, we applied time-lapse microscopy after the microinjection of an H2B-EGFP mRNA construct. Both the control and NCS-treated embryos formed correct chromosomes’ alignment in the metaphase in the same time period. Chromosomal segregation errors were more often visible in NCS-treated embryos during the prometaphase and metaphase. These chromosomal fragments were localized outside of the metaphase plate and persisted until the anaphase stage of the first cell cleavage (Figure 7 and Supplementary Data, Movie S1). As analyses revealed, the frequency of the chromosome segregation problems depended on the concentration of NCS applied in the G1-phase (Figure 8). Thus, segregated chromatin structures were also observed outside of the new formed nuclei in two-cell embryos.

### 2.6. Low Dose of NCS Does Not Affect Entry into Anaphase but Induces Micronuclei Formation

**Figure 9:**
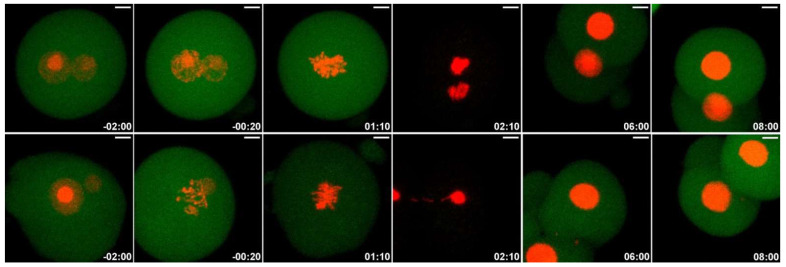
Still images from a time-lapse recording of the first mitosis in the control and NCS-treated one-cell embryo expressing securin-EGFP (green) and H2B-mCHERRY (red) mRNA. The Z-projections show the maximum intensity of the confocal sections. Scale bar = 10 µm. Additionally, see Movie S2 in the Supplementary Data.

**Figure 10:**
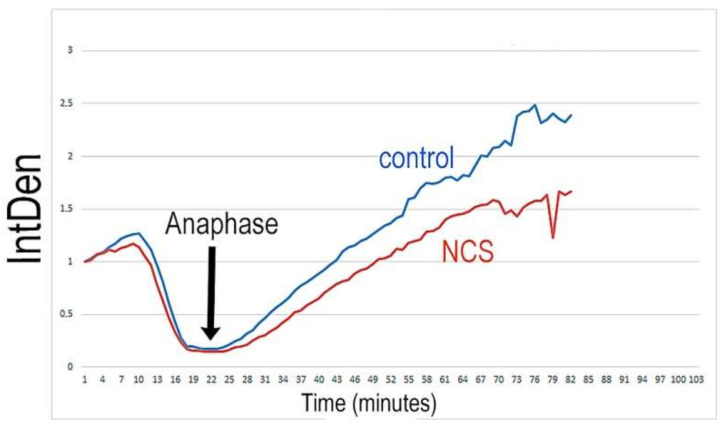
Time course of the changes in the level of securin during the first cleavage in control and NCS-treated embryos.The graph shows relativized values of the measured fluorescent signal densities.

Because NCS treatment in the G1-phase results in an increased number of γH2A.X^Ser139^ foci, we addressed the question as to whether anaphase-promoting complex/cyclosome (APC/C) is activated by the degradation of securin in low doses of NCS (20 ng/mL). In the control group of embryos, all the chromosomes were successfully aligned to the metaphase plate, and securin-EGFP destruction was visible immediately before the anaphase initiation of chromosome segregation. Securin was re-accumulated after the first division when new nuclei were formed. NCS-treated embryos display the same character of the process except for a very short delay in the start of the re-accumulation of securin in the two-cell stage. The analysis of the changes in the level of securin in real time documented the maximum fall of securin immediately before the onset of the anaphase in both the control and NCS-treated one-cell embryos (Figure 9 and Figure 10 and Supplementary Data, Movie S2) in addition to re-accumulation in two-cell embryos when new nuclei were formed. These data prove that low damage of DNA does not affect the course of cytokinesis in one-cell embryos at low levels of DNA damage.

**Figure 11:**
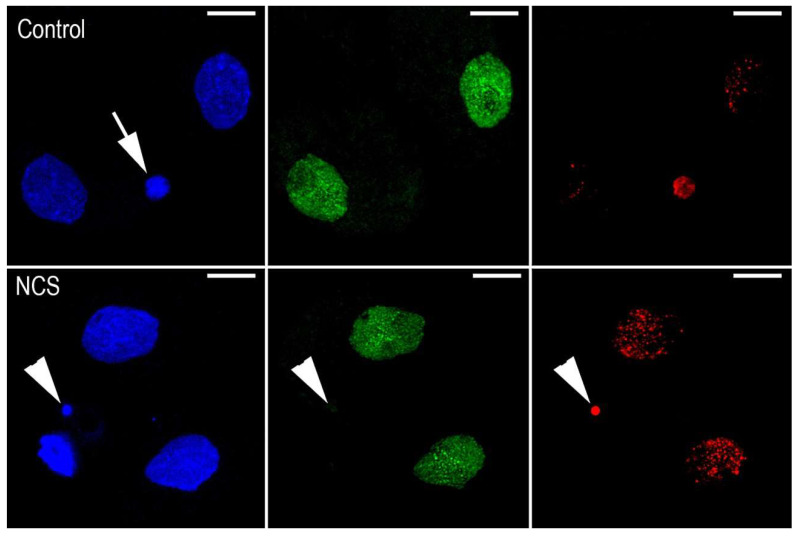
Double immunostaining of two-cell embryo after NCS treatment in the G1-phase of the one-cell stage. DAPI staining (blue) shows the nuclei and the micronucleus (arrowhead)positive forγH2A.X^Ser139^ (red fluorescence—arrowhead). The green immunofluorescence documents the incorporation of EdU into DNA of the nuclei only, but not in the micronucleus (green fluorescence—arrowhead). Polar body in the control embryo stained with DAPI (arrow). Scale bar = 10 µm.

**Figure 12:**
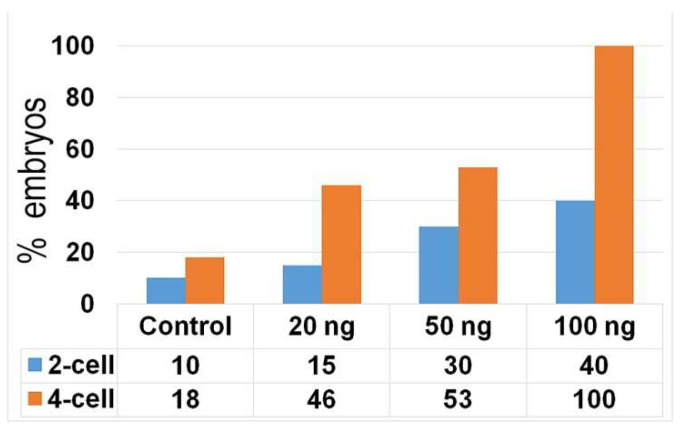
Percentage of the control and NCS-treated two- and four-cell embryos that contain micronuclei in at least one blastomere.

We observed the segregation of condensed chromosomes during the monitoring of chromosome dynamics in one-cell embryos that contained damaged DNA. When these embryos formed daughter nuclei of the two-cell stage, small fragments of condensed chromatin were detected to be segregated outside of the nuclei. These condensed chromatin fragments, designated as micronuclei (MNs), were detected in only one blastomere of two-cell or four-cell embryos. As expected, the immunofluorescent signal for the incorporation of an EdU pulse into DNA was detected in the nuclei of both the control and NCS-treated embryos. In contrast, the condensed segregated DNA fragments (micronuclei) were free of the EdU immunofluorescent signal. On the other hand, the same fragments that were positive for the γH2A.X^Ser139^ immunofluorescent signal were also observed also during the third cleavage period, and therefore, in the four-cell stage of preimplantation embryo (Figure 11). As documented by image analysis, the incidence of these fragments was higher in NCS-treated embryos, depending on the concentration of the drug. This analysis also showed increased incidence of these fragments in four-cell embryos in comparison with previous (two-cell) stages (Figure 12).

### 2.7. DNA Damage Increases Chromatin Fragmentation and Reduces the Number of Blastomeres

**Figure 13:**
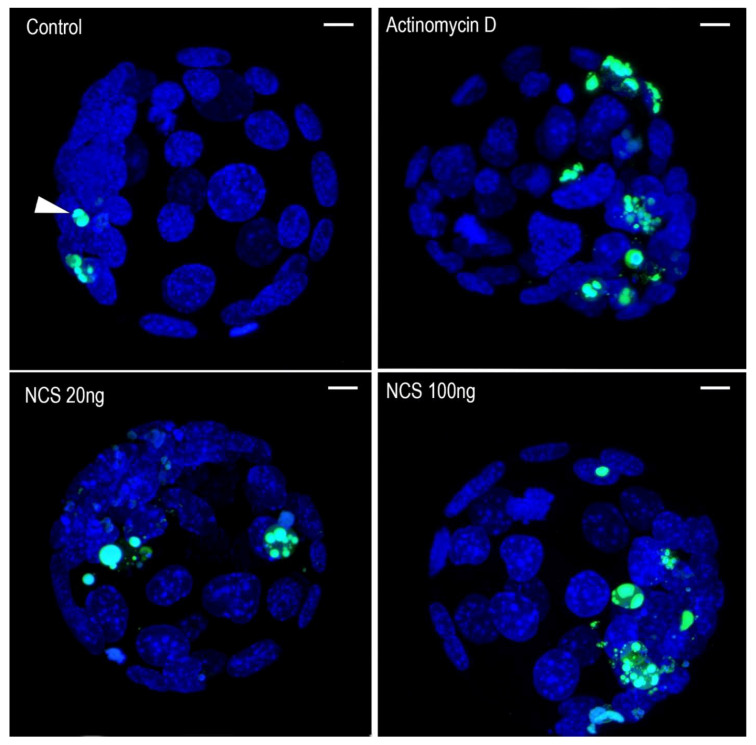
DNA degradation visualized by TUNEL staining (green—arrowhead) in mouse blastocysts. DAPI staining (blue) shows nuclei of blastomeres. That Z-stack projections of the serial confocal sections across whole blastocysts are shown. Scale bar = 10 µm.

**Figure 14:**
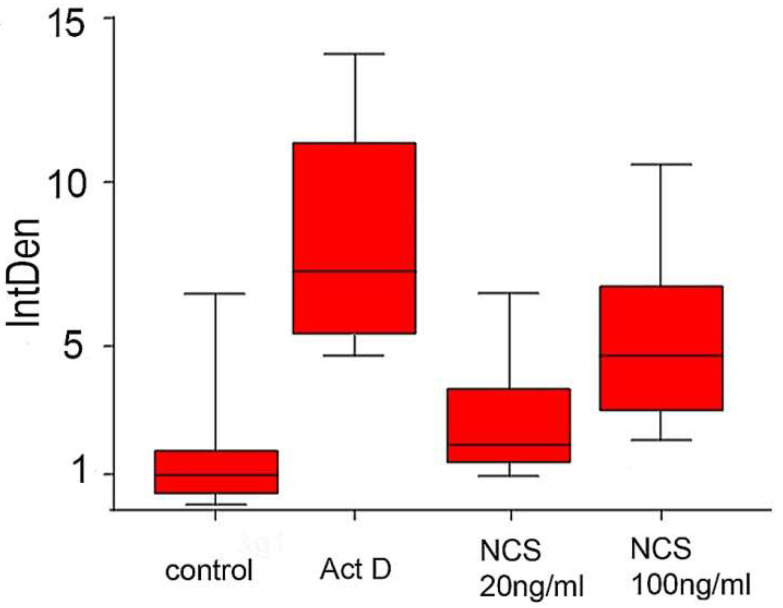
Quantification of TUNEL signal intensity in control, actinomycin D-treated blastocysts, and blastocysts after previous treatment with NCS during one-cell stage. The values show the average relative density of the fluorescent signal in the blastocyst of each experimental group.

Nuclear morphology visualized by chromatin staining with Hoechst 33342 revealed chromatin condensation or fragmentation in all (control and experimental) blastomeres. Chromatin changes defined in this way have in the majority been positive for TUNEL staining (Figure 13). The total TUNEL signal intensity measured in each blastocyst was highest in the actinomycin D-treated embryos and lowest in the control group. In the NCS-treated embryos, an increased TUNEL positive signal was related to an increased NCS concentration (Figure 14).

**Figure 15:**
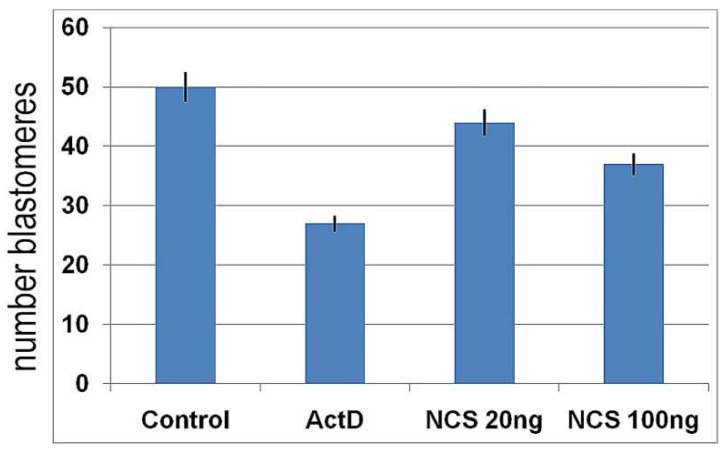
Average number of blastomeres per blastocyst in control, actinomycin D-treated blastocysts, and blastocysts after previous treatment with NCS during one-cell stage.

On the other hand, the average number of blastomeres in each blastocyst was highest in the control embryos and lowest in the actinomycin D-treated embryos. In the NCS-treated embryos, a decreased average blastomere count was related in the blastocysts that were treated with a higher concentration of NCS during their first cleavage stage (Figure 15).

## 3. Discussion

Our study demonstrates that DNA damage induced before the start of DNA synthesis in one-cell embryos can significantly affect the preimplantation development of the embryo. This developmental ability is related to the level of DNA damage. The results of the experiments reported here extend the previous finding that one-cell embryos can complete the first cleavage cycle despite incomplete replication after low DNA damage. We assume that this phenomenon creates a predisposition to a segregation disorder of condensed chromatin that results in the formation of micronuclei in future developmental stages following the first cleavage.

In the presented study we used the protein small molecule complex neocarzinostatin (NCS), which induces sequence-specific double-stranded breaks in DNA (DSBs), considered to be the most important type of DNA damage [25,26]. Although DNA damage can also be induced by other chemical drugs [22,27], we used NCS due to previous oocyte experimental models [18]. It should be noted that H2A.X phosphorylation, as a DNA damage marker, does not always depend on double-stranded breaks [28,29]. However, we found that inducing new DSBs by NCS results in a concentration-dependent increase in γH2A.X^Ser139^/MDC1-positive foci that is easily detected very shortly after the application of NCS in early embryo cultures in vitro. This made it possible to precisely quantify the numbers of these foci during the first cleavage stage.

It was previously documented that preimplantation embryos are sensitive to DNA damage induced previously in spermia, oocyte, or early cleavage stages [11,14,15,30,31,32,33]. In connection with the topic of DNA damage, the immunostaining of γH2A.X^Ser139^ foci is widely accepted as a sensitive marker for double-stranded breaks of DNA (DSBs) [34]. Phosphorylated histone H2A.X (γH2A.X^Ser139^) is well-detectable using affinity cytochemistry during all stages of embryo [19,35] and oocyte [18]. In our double-immunostaining approach we used the immunofluorescent detection of the MDC1 protein, which has been shown to interact with H2A.X near the sites of DNA double-stranded breaks and recruit ATM kinase to the damaged region of DNA [36]. Our analysis of the γH2A.X^Ser139^ foci counted during the first cleavage cycle documents differences between control embryos in the G1-phase and G2-phase. In addition, significant differences in the foci number are evident when comparing the control and NCS-treated embryos in which the concentration dependence of NCS is demonstrated. Separate measurements of the incidence in both the male and female pronuclei always showed higher values in the case of the male pronucleus. Such γH2A.X foci asymmetry was previously presented in mouse zygotes without induced DNA damage [37], and discussed as a pre-replicative stage in the pronucleus with respect to the timing of demethylation to be linked to the DNA repair process [4,38,39]. This asymmetry is in correlation with the fact that, during the first replication cycle, the egg and sperm chromosomes replicate separately before their fusion at the end of DNA synthesis to generate the embryo’s nucleus [40,41]. Based on our morphometric analyses, we can say that γH2A.X^Ser139^/MDC1 foci are induced by NCS before the resumption of DNA synthesis in one-cell mouse embryos as well as the fact that the incidence of the foci depends on the drug concentration.

It is generally known that the first DNA replication cycle in fertilized eggs occurs in the molecular environment, which is different from that of somatic cells and can greatly influence the response to genomic insults. A significant number of one-cell mouse embryos were able to enter the S-phase despite DNA damage in the previous G1-phase. These embryos synthetized a new DNA, albeit at a reduced rate compared with the control embryos. Finally, we showed that many embryos, even with a lower level of newly synthetized DNA, can continue successfully in the first cleavage process. Based on these data, it is clear that the efficiency of the G1/S checkpoint in one-cell mouse embryo has its limits in relation to the level of DNA damage. In this sense, the same situation was observed in the case of the G2/M checkpoint because the first cell cycle continued despite incomplete DNA replication. It was similar in the case of the SAC regulatory mechanism (spindle assembly checkpoint), which controls kinetochore microtubule attachment following the anaphase phase. Our results confirm the fact that early embryos containing mild DNA damage can overcome the SAC in the first cleavage stage in a similar way as in the following stages up to the blastocyst stage [40]. This makes it clear that DNA damage induced in a very early phase of the one-cell embryo stage is tolerated until later stages of embryo development and the one-cell mouse embryos can complete the first cleavage stage. This phenomenon was documented in human cancer cells and was defined as checkpoint adaptation to treatment with pharmacological genotoxic agents. In the context to of checkpoint adaptation, micronuclei formation and chromothripsis are biologically linked steps that contribute to genomic instability [42].

Chromosomal segregation errors observed in our NCS-treated embryos can most likely be attributed to the micronuclei (MNs) formation process. The MNs presented in this study were not active in DNA synthesis and were inherent in only one blastomere of two-cell embryos or four-cell embryos. Although the cellular impact of MNs formation is less understood, we propose that the occurrence of MNs in early embryos is related to DNA replication disorders following the incorrect attachment of spindle microtubules to kinetochores. As Vázquez-Diez et al. [43] presented previously, MNs can be inherited several times without rejoining the principal nucleus and without altering the kinetics of cell divisions. Thus, unilateral MNs inheritance can contribute to the high frequency of aneuploid cells in mammalian embryos, but simultaneously may serve to insulate the early embryonic genome from chromothripsis. Consistent with the conclusions of the observations of somatic cells we assume that micronuclei may persist in cells over several cleavage stages during early embryo development. On the other hand, γH2A.X^Ser139^-positive embryonal MNs showed no DNA replication activity in contrast to human somatic cells, in which reduced DNA replication in MNs was documented to be inefficient and asynchronous with the primary nucleus [44]. It is very likely that, the micronuclei occurring during early embryogenesis arise due to carryover of unresolved DNA damage into mitosis. In this context, it was shown that 85% of embryos containing micronuclei display a γH2A.XSer^139^-positive signal, which indicated increased DNA damage [23].

Despite extensive research on early embryos, all effects of gene instability on early embryo development are still not well-understood. A remaining question to be answered: what contributes to tissue and lineage-specific responses to DNA damage in blastocyst? Embryonic development is manifested by specific cellular/molecular dynamics that are different from those of somatic cells. These characteristics are more similar to those of cancer cells in terms of a high proliferation rate, increased replication stress and gene instability [2].

Embryonic cells can utilize different methods to overcome replication disorders resulting from DNA damage. There are effectively three options: The first option is the activation of apoptotic pathways, leading to cell destruction. The second is tolerance of the intracellular lesions, leading to possible mutations or later developmental disorders. The third option is the best because it activates repair mechanisms administered by so-called cell cycle checkpoints G1/S, G2/M, and SAC. However, the stage timing is controlled by maternally inherent mRNA during the first two to three cleavages, because early embryos activate their embryonal genome gradually. During the cleavage stages, from one-cell to blastocyst, the newly synthetized repair proteins are accumulated fully functional apoptotic machinery that can effectively remove damaged blastomeres [45]. This fact appears to be related to our observation of a reduced number of blastomeres in the blastocysts after DNA damage. The same phenotype was observed in connection with the formation of micronuclei after the application of the pesticide “chlorpyrifos“ [46].On the other hand, antiapoptotic proteins in early embryos are active to protect the cleavage of embryos in the processes of transformation into embryonal genome. However, developmental arrest occurs in the case of critical levels of damage [47]. Cleaving embryos tolerate DNA damage by suppressing apoptosis before the final preimplantation stages because due to a highly contracted cell cycle and a very short S-phase there is not enough time for repair. In addition, these early embryos tolerate a threshold level of the incorporation of ribonucleotides into the DNA, although it can initiate a response that leads to developmental disorders [48] and the possible depletion of some differentiated blastomeres from the inner cell mass [49]. Blastocysts after primary differentiation possess all the mechanisms necessary for the recognition, engulfment, and finally elimination of damaged blastomeres [50]. It was also demonstrated that an early chromosomes segregation error causing the formation of micronuclei affects ploidy and the development into blastocyst but does not necessarily cause developmental failure after the blastocyst stage [51]. In this context, the selection of embryos immediately before implantation seems to be very important. Later in development, mainly during the morula and blastocyst stages, apoptosis would mediate the elimination of certain cells, accomplishing both a physiological role in balancing cell proliferation and death, and a pathological role in preventing the transmission of damaged cells with an altered genome. However, despite the significant progress made in the field of assisted reproduction during the last decades, remains the case that more than 50% of the embryos produced in vitro do not reach the blastocyst stage [52]. Recently published data reveal that the presence of fragmented cells is not an indicator of blastocyst quality because these embryos can be successful in extruding abnormal cells in an effort to maintain their developmental potential. In this context, Yu et al. [53] showed that there is no correlation between the presence of cellular fragments in the perivitelline space and blastocyst quality. Even blastocysts with a good morphology will not necessarily implant and provide healthy offspring.

## 4. Materials and Methods

### 4.1. Embryo Collection and In Vitro Culture

**Figure 16:**
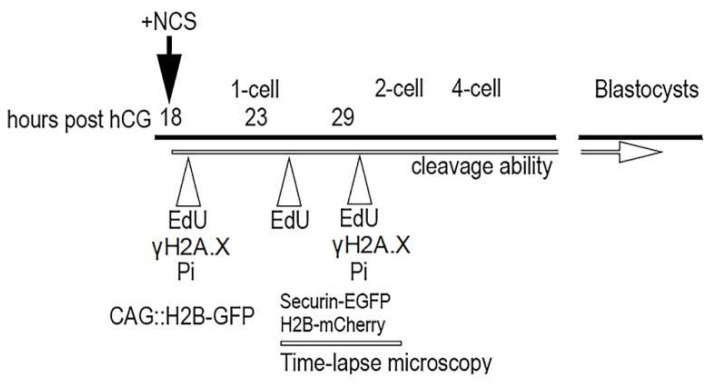
Experimental design. All one-cell embryos were collected at time 18 h post hCG administration and each experiment (as reported in Material and Methods) was performed separately in triplicate.

CD-1 IGS mice (Velaz Ltd., Praha, Czech Republic) 6 to 8 weeks of age were superovulated with 5 IU of pregnant mares’ serum gonadotropin (eCG, 5IU intraperitoneally) (Folligon, Intervet Int. BV, Boxmeer, Holland), followed 46-47 h later by 5 IU of human chorionic gonadotropin (hCG, 4IU intraperitoneally) (Pregnyl, Organon, Oss, The Netherlands), then mated in vivo with males. Fertilized one-cell embryos were isolated from the ampulla oviduct of plug-positive females 18 h post-hCG, and collected into a defined M2 or “HTF for murine IVF” medium (Millipore, Darmstadt, Germany). The cumulus cells were dispersed with hyaluronidase diluted (1 mg/mL) in the same medium. These embryos were followed immediately by culturing in vitro in defined KSOMaa Evolved medium (Millipore, Darmstadt, Germany) at 37 °C in humidified air containing 5% CO_2_. Thus, collected embryos were divided into (1) control embryos and (2) embryo treated with neocarzinostatin (NCS) (Sigma-Aldrich, St. Louis, MO, USA) subsequently managed according to the strategy of individual experiments. The experimental strategy was based on the evaluation of phenotypic changes in the embryos after the induction of DNA damage in the first cleavage cycle. DNA damage was induced by the radiomimetic drug NCS, which effectively induces sequence-specific double-stranded breaks in DNA. This inductor was applied to the KSOMaa medium immediately after the isolation of one-cell embryos from oviduct at the time 18 h post-hCG administration in concentration 20 ng/mL, 50 ng/mL or 100 ng/mL analogous to our previous experiments on mouse oocytes [18], for 60 min. The control embryos were cultured in a pure KSOMaa medium. In the baseline, the experimental strategy was divided into (1) the evaluation of the cleavage ability of early embryos; (2) the occurrence of double-immunostained DNA damage foci on γH2A.X^Ser139^ and MDC1; (3) the measurement of the volume of DNA in the pronuclei of late one-cell embryos; (4) the monitoring of anaphase-promoting complex/cyclosome (APC/C) activity during the first cytokinesis in real time and monitoring of condensed chromosomes during the first cytokinesis in real time; and (5) the analysis of the incidence of blastomeres with fragmented chromatin with a TUNEL assay and the total number of blastomeres in expanded blastocyst. In the pilot test, we determined more precise timing of the G1-phase, S-phase, and G2-phase during the first cleavage cycle in our mouse model. For the metabolic labeling of DNA we applied an EdU assay that detected the incorporation of ethynyl-deoxyuridine into DNA during DNA synthesis [54].The culture medium was supplemented with 5-ethynyl-2’-deoxyuridine (EdU) (Sigma-Aldrich, St. Louis, MO, USA) for 30 min in three separate phases during the first cell cycle at 18 h (G1-phase), 23 h (S-phase) or 29 h (G2-phase) post hCG administration and then immediately fixed in 3.7% paraformaldehyde (Sigma-Aldrich, St. Louis, MO, USA). AlexaFluor 488 (Jackson ImmunoResearch, Baltimore, MD, USA) was used for the staining of newly synthetized DNA. The designs of individual experiments are shown in Figure 16.

### 4.2. Evaluation of the Ability to Cleavage

The cleavage ability of the control and NCS-treated embryos inin vitro conditions was evaluated under an inverted microscope (Nikon-Eclipse Ti) at defined time intervals of 44, 67, 91 and 113 h post hCG administration.

### 4.3. Immunocytochemistry

In the pilot experiments we detected the timing of active DNA synthesis in our experimental mouse model via an EdU assay which detects the incorporation of the ethynyl-deoxyuridine into DNA [55]. Based on the results obtained in the EdU assay, the embryos cultured in vitro were then harvested 19 or 30 h post hCG administration (i.e., the G1-phase or G2-phase). After brief washing in phosphate-buffered saline (PBS), the embryos were fixed in 3.7% paraformaldehyde diluted in PBS for 50–60 min at room temperature (RT). After washing in PBS (3 min × 20 min), the embryos were permeabilized with 0.5% Triton X-100 in PBS for 45 min at RT and preincubated with inactive 2% normal donkey serum (Jackson ImmunoResearch, Baltimore, MD, USA) in PBS for 2 h at RT. For indirect immunofluorescence, the embryos were incubated overnight at 4 °C with rabbit primary antibody against γH2A.X^Ser139^ (Cell Signaling Technology, Danvers, MA, USA) diluted 1:200 in 0.3% normal donkey serum. For double-staining we used rabbit primary antibody against γH2A.X^Ser139^ (Cell Signaling Technology) and mouse primary antibody against MDC1 which interacts with γH2A.X^Ser139^ in sites of DNA double-stranded breaks (Millipore). After extensive washing, the embryos were stained with affinity purified secondary antibodies coupled with fluorescein (FITC) or rhodamine (TRITC) for 60 min at RT (Jackson ImmunoResearch, Baltimore, USA). The specificity of the immunostaining was established by omitting the primary antibodies or using another species-specific fluorescein conjugate. Finally, the DNA was counter-stained with DAPI.

### 4.4. Propidium Staining

In the experiment focused on the analysis of the volume DNA, a propidium iodide standard staining protocol (BioLegend, San Diego, CA, USA) was applied. The experimental embryos were fixed in 70% ethanol (for 5 min at −20 °C) and treated with RNase (Sigma-Aldrich, St. Louis, MO, USA). After a final washing in PBS, all the stained embryos were mounted in Mowiol medium.

All embryos were scanned using a Leica TCS SP5 with an HCX PI Apo Lambda Blue 63 × 1.4 oil objective. A sequential scan was applied in16-bit image depth with 1024 × 1024-pixel image resolution. Three-dimensional scanning was performed using 1 µm optical sections through the embryo volume.

### 4.5. Time-Lapse Recording

We monitored APC/C activity using the well-established marker securin-EGFP [24] expressed together with H2B-mCHERRY from microinjected mRNAs. APC/C activation mediates the degradation of securin and cyclin B to trigger the anaphase [16]. For the live imaging of chromosome dynamics during the first cycle, we used CAG::H2B-GFP transgenic mice [56]. In experiments focused on the monitoring of APC/C activity during cytokinesis in the first cleavage cycle we used vectors for securin-EGFP and H2B-mCherry [57] for in vitro mRNA transcription as we described on oocytes [58,59,60]. Fertilized embryos were microinjected after culture in medium supplemented with NCS and washing in pure culture medium. Concentrations of 20 ng/µL of securin-EGFP and 75 ng/µL of H2B-mCherry mRNAs in M2 medium were used at the time 20 h post hCG administration. Time-lapse image acquisitions were performed using a Leica TCS SP5 confocal system equipped with an acousto-optical beam splitter (AOBS) by applying the following settings: lens PlanNeoFluor 40 × 1.25 oil objective, 1024 × 1024 px resolution, 16-bit depth, 12 stacks with 7.4 µm thickness, time frame of 5 min. Lasers of 488 nm and 566 nm at 3% power were used for EGFP and mCHERRY excitation. An EGFP signal was detected at 500–560 nm, and an mCHERRY signal was detected at 580–630 nm. Image data were processed using FiJi Software [30]. Four-dimensional data (3D+time) were denoised using mean or median filters, and images were presented as maximum projection or as a single confocal section for brightfield. The time of nuclear envelope breakdown (NEBD) (time of mitosis resumption) was defined as the time when chromosomes began to be condensed (denoted as “0:00” in Figure 1 and Movie S1).

### 4.6. TUNEL Assay

DNA fragmentation was detected by terminal deoxynucleotidyltransferase dUTP nick end labeling using a DeadEndFluorometric TUNEL system (Promega Corp., Madison, WI, USA). In the same blastocysts, cell nuclei were counter-stained with Hoechst 33342 (20 mg/mL, Sigma-Aldrich, Missouri, USA and finally mounted with Vectashield (Vector Laboratories, Burlingame, CA, USA) on glass slides. In the positive control group, actinomycin D (as a standardized apoptosis inductor) was applied in concentration 5 ng/mL for 22 h in the culture time interval of 91 h post hCG. All blastocysts were analyzed for their number of blastomeres as well as for the intensity of the fluorescence signal on the chromatin fragments and chromatin condensed bodies in the time culture interval of 113 h post hCG administration.

### 4.7. Image Analysis

All processed samples were scanned using a Leica TCS SP5. As the sequencing mode, an Apo Lambda Blue 63 × 1.4 oil objective was used and individual 1 µm optical sections were recorded in 12-bit depth with 1024 × 1024-pixel resolution on each embryo. Three-dimensional scanning was performed through the embryo volume.

An image analysis was performed using the FiJi Software. In the case of DNA damage analysis γH2A.X^Ser139^–positive foci and TUNEL-positive signals we segmented the fluorescence signal by applying an intensity threshold on 3D images previously denoised using the PureDenoise plugin followed by a 3D Object Counter measurement [61,62].

## 5. Conclusions

We can conclude that one-cell mouse embryos tolerate a certain degree of induced DNA damage before entry to the S-phase considers it a priority to complete the first cleavage stage and continue embryogenesis as far as possible.

## Data Availability

All data are available at the Institute of Animal Physiology, Centrum of Biosciences, Šoltésovej 4-6, 040 01 Košice, Slovakia (Vladimir Baran, baran@saske.sk), and also Institute of Animal Physiology and Genetics of the Czech Academy of Sciences, Liběchov, Czech Republic (Dávid Drutovič, drutovic@iapg.cas.cz).

## Supplementary Materials

The following supporting information can be downloaded at: https://www.mdpi.com/article/10.3390/ijms23073516/s1.

## Abbreviations

APC/C	Anaphase-promoting complex/cyclosome
DAPI	4’,6-diamidino-2-phenylindole
DSBs	Double-stranded breaks
eCG	Pregnant mare’s serum gonadotropin
EdU	5-ethynyl-2’-deoxyuridine
EGFP	Enhanced green fluorescent protein
FITC	Fluorescein.5-isothiocyanate
hCG	Human chorionic gonadotropin
MN/MNs	Micronuleus/Micronuclei
NCS	Neocarzinostatin
NEBD	Nuclear envelope breakdown
PBS	Phosphate buffer saline
RT	Room temperature
SAC	Spindle assembly checkpoint
TRITC	Tetramethylrhodamine
