# Correction: Baran et al. Cleavage of Early Mouse Embryo with Damaged DNA. *Int. J. Mol. Sci.* 2022, *23*, 3516

**DOI:** 10.3390/ijms26052171

**Published:** 2025-02-28

**Authors:** Vladimír Baran, Tomáš Ďuríček, Jozef Pisko, Dávid Drutovič, Petr Šolc

**Affiliations:** 1 Institute of Animal Physiology, Centre of Biosciences, Slovak Academy of Sciences, Šoltésovej 4, 040 00 Košice, Slovakia; pisko@saske.sk; 2 Institute of Animal Physiology and Genetics of the Czech Academy of Sciences, Rumburská 89, 277 21 Liběchov, Czech Republic; tom.duricek@gmail.com (T.Ď.); drutovic@iapg.cas.cz (D.D.); 3 Department of Cell Biology, Faculty of Science, Charles University, Viničná 7, 128 00 Prague, Czech Republic

In the original publication [1], the following three authors were not included. The newly included authors (Tomáš Ďuríček, Dávid Drutovič and Petr Šolc) were involved in conceptualization, investigation and supervision. The affiliations, author contributions statement and funding sections have been corrected accordingly.

The authors state that the scientific conclusions are unaffected. This correction was approved by the Academic Editor. The original publication has also been updated.

## Data Availability

All data are available at the Institute of Animal Physiology, Centrum of Biosciences, Šoltésovej 4-6, 040 01 Košice, Slovakia (Vladimir Baran, baran@saske.sk), and also Institute of Animal Physiology and Genetics of the Czech Academy of Sciences, Liběchov, Czech Republic (Dávid Drutovič, drutovic@iapg.cas.cz).
